# Suspected Adverse Drug Reactions Related to Breast Cancer Chemotherapy: Disproportionality Analysis of the Brazilian Spontaneous Reporting System

**DOI:** 10.3389/fphar.2019.00498

**Published:** 2019-05-08

**Authors:** Flávia Campos Barcelos, Guacira Corrêa de Matos, Mario Jorge Sobreira da Silva, Fabrício Alves Barbosa da Silva, Elisangela da Costa Lima

**Affiliations:** ^1^Brazilian National Cancer Institute (INCA), Rio de Janeiro, Brazil; ^2^Faculty of Pharmacy, Federal University of Rio de Janeiro, Rio de Janeiro, Brazil; ^3^Oswaldo Cruz Foundation (Fiocruz), Rio de Janeiro, Brazil

**Keywords:** pharmacovigilance, oncology, disproportionatily analysis, spontaneous reporting system, adverse drug reaction

## Abstract

Spontaneous reporting systems may generate a large volume of information in real world conditions with a relatively low cost. Disproportionality measures are useful to indicate and quantify unexpected safety issues associated with a given drug-event pair (signals of disproportionality), based upon differences compared to the background reporting frequency. This cross-sectional study (2008 to 2013) aimed to analyse the feasibility of detecting such signals in the Brazilian Pharmacovigilance Database comprising suspected adverse drug reactions related to the use of doxorubicin, cyclophosphamide, carboplatin, trastuzumab, docetaxel, and paclitaxel for breast cancer chemotherapy. We first accessed overall database features (patient information and suspected adverse drug reactions) and further conducted a disproportionality analysis based on Reporting Odds Ratios with a confidence interval of 95% in order to identify possible signals of disproportionate reporting, only among serious suspected adverse drug reactions. Of all data reports of adverse reactions (*n* = 2603), 83% were classified as serious, with the highest prevalence with docetaxel (78.1%). The final analysis was performed using 1,309 reports with 3,139 drug-reaction pairs. The following signals of disproportionate reporting, some rare or not mentioned on labels, were observed: tachypnea with docetaxel; bronchospasm, syncope, cyanosis, and anaphylactic reaction with paclitaxel; and anaphylactic shock with trastuzumab. Structured management of spontaneous adverse drug reaction reporting is essential for monitoring the safe use of drugs and detecting early safety signals. Disproportionality signal analysis represents a viable and applicable strategy for oncology signal screening in the Brazilian Pharmacovigilance Database.

## Introduction

Pharmacovigilance activities—aimed at monitoring the safe use of medicines—are particularly important in oncology due to the inherent toxicity of antineoplastic agents (Baldo and De Paoli, 2014). Given the high incidence, prevalence and mortality of breast cancer in the worldwide female population (International Agency for Research on Cancer., 2014), knowledge of the toxicity profile of the main drugs used in its treatment is important strategically for prevention, detection and early management of suspected adverse drugs reactions (ADRs) related to chemotherapy.

Spontaneous reporting of ADRs contributes substantially to signal detection in drug safety surveillance, especially for rare and acute reactions (Arnaud et al., 2017). In pharmacovigilance, a signal is reported information on a possible causal relationship between an adverse event and a drug exposure, which was previously unknown or incompletely documented. It also refers to an increased number adverse events compared to the frequency of reactions normally expected with the use of a given product (U.S. Department of Health and Human Services et al., 2005; World Health Organization (WHO)., 2018).

Reports of ADR screening from the national surveillance system may offer data pertaining to different drug-event combinations (or drug-event pairs). These include methods of disproportionality analysis (DPA) that represent the main class of analytical methods for spontaneous report systems (SRS) data analysis in pharmacovigilance (Harpaz et al., 2012; European Medicines Agency., 2016)

Such methods, aimed at drawing attention to unexpected associations by generating hypothesis, have been guiding pharmacovigilance experts from health agencies worldwide in their investigations to draw definitive conclusions (Courtois et al., 2018).

Quantitative signal detection methods for spontaneously reported data include Proportional Reporting Ratio (PRR), Reporting Odds Ratio (ROR), and Empirical Bayes Geometric Mean (EBGM). These methods may identify relevant associations in SRS databases, focusing on projections of lower data dimensionality, more specifically two-dimensional contingency tables. Frequentist approaches are usually accompanied by independence hypothesis tests (Bate and Evans, 2009).

Brazilian Reporting System in Health Surveillance (Notivisa) was created in 2008. Since then, no evaluations of signal detection through disproportionality analysis have been made using this national database (Mota et al., 2018).

This study aimed to analyse serious ADRs associated with the most commonly used drugs in first-line adjuvant chemotherapy for breast cancer, reported from the Brazilian Surveillance System, in order to verify the feasibility of identifying potential signals through DPA using this database.

## Materials and Methods

We carried out a cross-sectional study related exclusively to spontaneous ADRs reported in Notivisa from 2008 to 2013 during breast cancer treatment with doxorubicin, cyclophosphamide, carboplatin, trastuzumab, docetaxel, and paclitaxel—which are the preferred regimens according to National Comprehensive Cancer Network and Brazilian guidelines (National Comprehensive Cancer Network., 2017; Brasil, 2018).

Notivisa is a computerized system developed by the National Health Surveillance Agency (Anvisa) to receive notifications of incidents, adverse events and technical complaints related to the use of products and services under sanitary surveillance (Mota et al., 2018; National Health System Surveillance (Anvisa)., 2018). Anvisa supplied data on the Notivisa website reports (Pharmacovigilance Module) as a Microsoft Excel® file, which was made available by collaboration research. The database provided did not allow patient identification. The study was approved by the Research Ethics Committee of the Federal University of Rio de Janeiro.

Suspected ADRs were analyzed for their origin (geographical location), the patient‘s age (in years) and suspected drug (Anatomical Therapeutic Chemical Classification System), and were also classified according to severity and System Organ Class (SOC/MedDRA). The findings were also compared to the information contained in drug labels (MedDRA, 2018).

We accessed only serious ADR reports by using the signals of disproportionate reporting (SDR) method and thresholds recommended by the European Medicines Agency (EMA), namely Reporting Odds Ratio (ROR) and thresholds based on the 95% confidence interval and number of individual cases (European Medicines Agency., 2016).

DPA methodologies use frequency analysis of two-dimensional contingency tables to estimate surrogate measures of statistical association between specific drug–event combinations mentioned in databases of spontaneous reports. For instance, the Reporting Odds Ratio (ROR) measure is defined by the formula below:

$$\begin{array}{l} \text{ROR}=\left(\text{a}.\text{d}\right)/\;\left(\text{c}.\text{b}\right) \end{array}$$

Where:

- The value “a” indicates the number of individual cases that list the target drug **P** and the target ADR **R**;
- The value “b” indicates the number of individual cases that list the target drug **P** but not the target ADR **R**;
- The value “c” indicates the number of individual cases that list the target ADR **R** but not the target drug **P**;
- The value “d” indicates the number of individual cases that do not list the target ADR **R** or the target drug **P**

Eudravigilance adopts the following criteria to define an SDR (European Medicines Agency., 2016):

- The lower bound of the 95% confidence interval of the ROR measure is >1;
- The number of individual cases (value “a”) is greater than or equal to 3 for active substances contained in medicinal products, included in an additional monitoring list defined by the European Medicines Agency;
- The number of individual cases is greater than or equal to 5 for the other active substances;
- The event belongs to the Important Medical Event Terms (IME) list, as defined by EudraVigilance.

In this work, we adopted a conservative approach: ROR calculations were performed for all drugs with ***a*** ≥3, and defined the concept of *SDR intensity* as a measure directly related to the value ‘a'. Therefore, we differentiated between drug-event pairs with ***a*** ≥ 5 (higher intensity) and pairs with 3 ≤ ***a*** < 5 (lower intensity).

A situation occurs when ***c*** = 0, i.e., when all database reports containing a specific suspected ADR are associated to only one drug. In this case, the ROR cannot be computed and the value of the ROR is arbitrarily set at 99.9 to reflect the presence of a possible SDR, according to EudraVigilance (European Medicines Agency., 2016).

We adopted the same approach in this work. The observed signals were listed and further classified according to their intensity and ADR frequency, described in drug labels as: very common, not common, rare or very rare.

## Results

Overall analysis was performed on 1,309 reports with 3,139 drug-event pairs (4% of total Brazilian reports for all medicines in the study period).

All reports came exclusively from nine out of the 27 Brazilian States (Rio de Janeiro, São Paulo, Minas Gerais, Rio Grande do Sul, Santa Catarina, Paraná, Mato Grosso, Mato Grosso do Sul e Bahia). Most (64.7%) were from hospitals located in the state of Rio de Janeiro. The median age of the female patients was 51.7 years (range: 25–87 years).

Around 83% (n = 2603) of total suspected ADR (drug-reaction pairs) were classified as serious, for causing: death (*n* = 6), life-threatening conditions (*n* = 79), hospitalizations (*n* = 57), permanent disabilities (n = 19) and medically important events (*n* = 2472). Of all serious ADRs, the majority (78.1%) were associated with docetaxel, with the greatest prevalence of those classified as general disorders (Table 1).

**Table 1:** Serious ADRs related to breast cancer chemotherapy by system organ groups (Brazilian Health System Surveillance, 2008–2013).

**Drugs**	**Serious ADRs (%)**	**Main ADR reporting groups- SOC/MedDRA (n)**				
		**General disorders**	**Vascular disorders**	**Respiratory disorders**	**Muscular disorders**	**GI disorders**
Docetaxel	2032 (78, 1)	750	480	395	289	147
Paclitaxel	240 (9, 2)	37	51	45	21	8
Doxorubicin	136 (5, 2)	36	22	8	0	14
Cyclophosphamide	43 (1, 6)	3	4	12	0	2
Trastuzumab	137 (5, 3)	65	18	7	11	7
Carboplatin	15 (0, 6)	3	1	3	0	4
Total	2603 (100)	894	576	470	321	182

*ADRs, suspected adverse drug reactions; SOC, System Organ Class; MedDRA, Medical Dictionary for Regulatory Activities; GI, gastrointestinal*.

### Disproportionality Analysis

SDRs of higher intensity (a ≥ 5) and lower intensity (a ≥3 and a < 5; a ≥ 3 and c = 0^*^) were observed for docetaxel, paclitaxel, trastuzumab, doxorubicin and cyclophosphamide. The disproportionality analysis did not identify any SDR for carboplatin.

Unexpected events considered rare, not common or not even mentioned on their respective drug labels and also known as acute and life-threatening were identified. The following are noteworthy: tachypnea with docetaxel, bronchospasm, anaphylactic reaction, cyanosis and syncope with paclitaxel and anaphylactic shock with trastuzumab (Table 2).

**Table 2:** Disproportionality analysis conducted on Notivisa (Brazil) data according to the methods recommended by EudraVigilance for routine signal detection.

**Drug**	**ADR**	**ROR (lower bound of the 95% CI)**	**SDR Intensity**	**ADR frequency on drug labels**
Docetaxel	Dyspnea	2.15	a ≥ 5	Very common
	Abdominal pain	2.39		Common
	Back pain	7.97		Common
	Thoracic pain	5.39		Common
	Flushing	4.94		Common
	Oral Discomfort	—	a ≥ 3 and c = 0^*^	Not Mentioned
	Throat pain	—		Not Mentioned
	Dry throat	—		Not Mentioned
	Bone pain	—		Common
	Scintillating scotomas	—		Rare#
	Blurred vision	—		Rare#
	Lipothymia	—		Not Mentioned
	Mouth paresthesia	—		Not Mentioned
	Tachypnea	—		Not Mentioned
	Somnolence	—		Not Mentioned
Paclitaxel	Allergy (unspecific)	15.11	a ≥ 5	Common
	Hypersensitivity	45.27		Common
	Hypertension	5.44		Common
	Hypotension	30.25		Very Common
	General discomfort	6.70		Rare
	Bronchospasm	14.94	a ≥ 3 and a < 5	Not Mentioned
	Anaphylactic reaction	14.94		Rare
	Cyanosis	9.95		Not Mentioned
	Myalgia	7.99		Very Common
	Syncope	4.97		Not Mentioned
Trastuzumab	Chills	102.69	a ≥ 5	Very Common
	Hypertension	2.24		Common
	Rash	4.63		Very Common
	Tremor	49.40		Not Mentioned
	Headache	4.09	a ≥ 3 and a < 5	Very Common
	Anaphylactic shock	—	a ≥ 3 and c = 0^*^	Not Common
Doxorubicin	Neutropenia	10.77	a ≥ 5	Very Common
	Nausea	4.50		Very Common
	Erythema	17.27		Common
	Pruritus	16.95		Common
	Phlebitis	—		Common
Cyclophosphamide	Neutropenia	47.03	a ≥ 5	Common
	Nasal discomfort	191.85	a ≥ 3 and a < 5	Not Mentioned
	Successive sneezing	—	a ≥ 3 and c = 0^*^	Not Mentioned

*SDR, Signals of Disproportionate Reporting (lower bound of the ROR 95% confidence interval >1); ROR, Reporting Odds Ratio (with 95% confidence interval calculated for each drug-reaction pair of serious ADRs in comparison to drug labels information)*.

* *ROR measure for a≥3 and c = 0 defined arbitrarily as 99.9 in order to reflect the presence of a possible SDR*.

*#Included in post-marketing period*.

## Discussion

The overall analysis in Notivisa has identified the reported prevalence of serious suspected ADRs with docetaxel and paclitaxel, mostly due to infusion-related reactions (IRR) that usually present with: flushing, rash, pruritus, fever, tremor, rigor, dyspnea, chest/back pain, nausea, light hypotension and tachycardia. Nevertheless, these sets of reactions tend to be mild to moderate and also common, occurring during the first few minutes of a first or a second drug infusion (Picard and Castells, 2015).

In fact, severe hypersensitivity reactions with taxanes are considered rare (incidence 3–5%) but have the potential to evolve rapidly toward a high risk scenario, characterized by a significant drop in blood pressure (systolic ≤ 90 mmHg) and/or syncope, bronchospasm, oxygen desaturation and anaphylaxis, which requires immediate therapeutic intervention (Brown, 2004; Picard, 2017).

The analyzed data indicated that careful monitoring of such reactions likely should have been done in Brazilian breast cancer patients, in the view of the large number of suspected ADRs related to docetaxel reported to the Notivisa. However, such high prevalence must be observed with caution due to the fact that the study time frame (2008–2013) corresponds exactly to the period immediately after the Sanofi-Aventis patent of Taxotere (docetaxel) expired in Brazil. This scenario might have influenced many health professionals and also pharmaceutical companies toward notification of suspected ADRs, due to the entrance of new generic brands of docetaxel into the Brazilian market. In addition, patients and health professionals often believe that generic cancer drugs have less quality, effectiveness and safety than branded-name drugs, leading to continuous attention and a higher tendency to report ADRs (Yang et al., 2016). It is also common to note that ADRs and therapeutic failure reports significantly increase after the entry of generic oncology drug into the marketplace (Pitts et al., 2016).

As trastuzumab, a monoclonal antibody, is also highly associated with IRR, symptoms such as fever, chills and tremor that were observed in Notivisa are considered common and may affect up to 40% of patients. However, urticaria, angioedema, anaphylaxis and anaphylactic shock resulting in hospitalization or death are quite infrequent (Lenz, 2007).

We also noticed that serious ADRs (cardiovascular, gastrointestinal and neurologic events) have been scarcely reported in Notivisa, despite being widely observed among patients undergoing chemotherapy for breast cancer with the studied drugs, both in the literature and in VigiAcces®–the free access platform of the UMC/WHO database VigiBase (Barbour, 2008; De Lullis et al., 2015; Frise et al., 2017; Martel et al., 2017).

Despite its importance in terms of drug safety monitoring, pharmacovigilance activities remain a challenge, mostly due to under-reporting. This common problem especially impacts the field of oncology due to the fact that ADRs are often considered “normal” (or inevitable) in cancer treatment. Additionally, the sensitivity and availability toward spontaneously reporting suspected ADRs can also vary between different kinds of health professionals and health systems all around the world (Baldo and De Paoli, 2014).

Therefore, using disproportionality measures, a quick, inexpensive and sensitive method of signal screening, has benefits and strengths in that it can provide valuable information on ADRs of greater clinical importance and higher risk in oncology (Montastruc et al., 2011; Dias et al., 2014; Tuccori et al., 2015).

In our study we managed to identify SDRs for docetaxel, paclitaxel, trastuzumab, and less frequently, for doxorubicin and cyclophosphamide, but not for carboplatin. The following drug-event pairs should be highlighted: allergy/ hypersensitivity, anaphylactic reaction and bronchospasm with paclitaxel and classical IRRs with docetaxel and trastuzumab.

In addition, DPA analysis could also identify some important drug-event pairs, whose ADRs were not clearly mentioned on labels or were considered uncommon in clinical trials, such as: docetaxel and tachypnea; paclitaxel and bronchospasm (not mentioned in label, but related to severe RRI); paclitaxel and anaphylactic reaction; paclitaxel and cyanosis; paclitaxel and syncope (not mentioned in label, but related to severe IRR); trastuzumab and tremor and trastuzumab and anaphylactic shock (both related to IRR).

However, such results should be interpreted with caution due to specific limitations of reporting system databases, with which it is not possible to ascertain causality. Thus, a relative increase in the proportion of notifications of a given drug-event pair may in fact be a false positive without any kind of causal relationship, particularly in cases with low report numbers, where the statistical disproportionality may reflect one or more biases (Dias et al., 2014; Hauben et al., 2017).

In addition, it is relevant to mention that some external factors, such as: (i) time on the market (new or old drugs); (ii) tendency to report only severe adverse events and (iii) selective reporting for a given drug may affect the reliability of detected disproportionality signals (Bate and Evans, 2009; Arora et al., 2017). These important points seem also to justify the great number of docetaxel reports in Notivisa (2008–2013).

Based on our findings, and in accordance with the literature, it would be possible to select specific events identified and investigate their relationship to all drugs (event-based approach) or to select specific drugs to monitor and their relationship with all possible events (drug-based approach) (Bate and Evans, 2009; Trifirò et al., 2009; Dias et al., 2014).

The study limitations result from characteristics related to the Brazilian Surveillance System Database (Notivisa) and other SRSs (biases of under-reporting, heterogeneity and selectivity), limits of DPA itself and the fact that the majority of suspected ADRs originated from only one Brazilian State. This last limitation seems to be due to the fact that one of the most important cancer treatment centers (National Cancer Institute/INCA—considered a reference standard in the treatment of breast cancer in Brazil) is located in the Rio de Janeiro State.

Finally, it must be said that data mining methods in pharmacovigilance are considered complementary and not substitutes for traditional signal identification strategies. It is necessary to evaluate, in advance, the accuracy of the signaling criteria used, the nature and the number of drugs and warning events to monitor, the potential impact of false positives or false negatives and the availability of resources (Bate and Evans, 2009; Montastruc et al., 2011; Hauben et al., 2017).

Nevertheless, our analysis indicates a potential use of Notivisa for signal detection in regards to clinical or regulatory applications, as it was able to identify relevant disproportionate signals considered severe and rare, and not mentioned on drug labels.

To our knowledge, this was the first study to apply DPA methods to the Brazilian surveillance database system (Notivisa).

## Conclusion

Our analysis using Notivisa showed a predominance of serious ADRs in regards to docetaxel and paclitaxel, as well as a greater tendency to report general, vascular and respiratory disorders, mostly related to IRR.

The DPA applied was able to identify some interesting signals worthy of further investigation related to antineoplastic agents in Notivisa, suggesting that such a method might be useful for other drug classes.

Despite its inherent limitations, SRS seems to benefit from this kind of approach, as it can potentially contribute to research, surveillance and the ever safer use of medicines in low and medium-income countries such as Brazil.

## Author Contributions

FCB, ECL, and GCM conceived and designed the study. FABS contributed to the disproportionality analysis. GCM and ECL contributed to the acquisition and the interpretation of data for the work. FCB, ECL, and MJSS discussed the results. FCB drafted the manuscript with support from ECL and MJSS. All authors critically revised the work and approved the final manuscript.

### Conflict of Interest Statement

The authors declare that the research was conducted in the absence of any commercial or financial relationships that could be construed as a potential conflict of interest.
